# Temporal stability of objective structured clinical exams: a longitudinal study employing item response theory

**DOI:** 10.1186/1472-6920-12-121

**Published:** 2012-12-07

**Authors:** Lubna A Baig, Claudio Violato

**Affiliations:** 1 Medical Education, College of Medicine, King Saud bin Abdulaziz University for Health Sciences, Riyadh, Saudi Arabia; 2 Medical Education Research Unit, University of Calgary, Calgary, Canada; 3 Medical Education Research Unit Faculty of Medicine, University of Calgary, Calgary, Canada

**Keywords:** Stability of OSCEs, Latent trait analyses, Ep^2^, Internal consistency

## Abstract

**Background:**

The objective structure clinical examination (OSCE) has been used since the early 1970s for assessing clinical competence. There are very few studies that have examined the psychometric stability of the stations that are used repeatedly with different samples. The purpose of the present study was to assess the stability of objective structured clinical exams (OSCEs) employing the same stations used over time but with a different sample of candidates, SPs, and examiners.

**Methods:**

At Time 1, 191 candidates and at Time 2 (one year apart), 236 candidates participated in a 10-station OSCE; 6 of the same stations were used in both years. Generalizability analyses (Ep^2^) were conducted. Employing item response analyses, test characteristic curves (TCC) were derived for each of the 6 stations for a 2-parameter model. The TCCs were compared across the two years, Time 1 and 2.

**Results:**

The Ep^2^ of the OSCEs exceeded.70. Standardized thetas (*θ*) and discriminations were equivalent for the same station across the two year period indicating equivalent TCCs for a 2-parameter model.

**Conclusion:**

The 6 OSCE stations used by the AIMG program over two years have adequate internal consistency reliability, stable generalizability (Ep^2^) and equivalent test characteristics. The process of assessment employed for IMG’s are stable OSCE stations that may be used several times over without compromising psychometric properties.

With careful security, high-stakes OSCEs may use the same stations that have high internal consistency and generalizability repeatedly as the psychometric properties are stable over several years with different samples of candidates.

## Background

The objective structure clinical examination (OSCE) has been widely used to assess knowledge, skills, attitudes, communications and professionalism in health professions since the early 1970s. The OSCE is considered a flexible approach for administering tests since it may include various assessment methods that target a broad set of clinical competencies. In a typical OSCE candidates rotate through a series of 5–20 minute stations performing brief standardized tasks [1,2]. OSCEs may include various types of assessment methods for the different stations ranging from procedural skills demonstrated on cadavers, simulators, or standardized patients (SPs), to multiple choice tests. Raters evaluate the examinees using specific content checklists for the different tasks and/or global rating scales for overall performance on a particular task. The OSCE is a performance-based assessment tool with some objectivity that concentrates mainly on skills, and to a lesser degree on knowledge [3]. The attributes measured through the OSCE include the ability to take a proper and adequate history, complete a physical examination, communicate effectively, manage patients’ health, interpret clinical results, and synthesize information [4,5]. The OSCE has been used for formative and summative evaluation of health professionals, including physicians, nurses and others [6].

There has been a great deal of research in the past 30 or so years investigating the validity and reliability of the OSCE [7]. Notwithstanding this work, no systematic research has been conducted on the psychometric stability of OSCE stations that are used repeatedly with different samples (e.g., medical students at different years). It is common practice to use the same OSCE stations repeatedly over time. Aside from issues of security, this practice raises issues of stability over time of the stations (i.e., temporal reliability).

The use of OSCEs to assess clinical competency of medical students began in the early 1980s [8]. The OSCE format and structure has evolved and changed over time and are now considered to be the most reliable tool for assessing clinical competence. High stakes examinations conducted for licensing local and international medical graduates (IMGs) in United States, United Kingdom and Canada utilize the OSCE format for assessing clinical competence [9,10]. The OSCE has been used for high stake exams like the College of Family Physicians of Canada (CFPC) and Medical Council of Canada (MCC) since the early 1990s, and are now used with the United States Medical Licensing Examinations (USMLE).

The major research focus in the use of OSCE has been in assessing and improving reliability, with slightly less emphasis on validity [11,12]. The majority of the research on the reliability of the OSCE has focused on sources of errors due to SPs, raters, checklists, global rating scales, case length, number of SP encounters and content sampling. Boulet et al [11]. have suggested that the SP that portrays the case may have a large impact on the reliability. In a large scale study, however, Tamblyn et al [13]. concluded that error due to SP portrayal can be reduced to negligibility with adequate training of the SPs (also Adamo^9^). Another common source of error in OSCEs is examiner inconsistency (i.e., poor inter-rater reliability). Downing [14] has argued that the largest source of errors is due to examiner inconsistency and that examiner training is required to reduce this error.

There are very few studies that have examined the stability of the stations over time. Hodges did, however, report that the reliability (internal consistency) of stations increased by α = 0.05 on reusing the stations with good psychometric properties (e.g., internal consistency) [15]. In a large scale study of 5,335 international medical graduates, McKinely and Boulet [16] studied score drift in a high-stakes performance-based assessment with 13,000 individual SP-examinee encounters over the 4 quarters during a one year period. They found that there were changes in the difficulty (i.e., means) of matched encounters over time. The effect sizes (d; standardized differences between matched encounters) ranged from −0.51 to 0.48 with a mean d = 0.08. Their findings suggest that the difficulty of the task is not stable although the mean d is small. McKinely and Boulet [16] concluded that the score drift for SP-based exams (e.g., OSCEs) needs to be monitored over time to determine whether cases become more difficult, easier or remain stable over several administrations. These researchers focused only on mean scores of the encounters, however, and did not investigate other properties of the assessments such as discrimination.

More advanced techniques such as item response theory (IRT) employing the many facet Rasch model (MFRM) have been employed for assessing the independence of items in chiropractic and nursing OSCEs [17]. This study, however, employed only a single parameter (item difficulty) in the application of IRT and did not study the temporal stability of the OSCE. Similarly, while the MFRM analyses has proven useful for monitoring and improving the quality of an OSCE with residents, there was no analyses of temporal stability [18]. The IRT approach has been broadly employed for assessing the psychometrics of paper based exams [19] but has not been applied to assessing the temporal stability of OSCEs.

A two-parameter model (difficulty and discrimination) has not been employed to assess the temporal stability of performance based examinations such as the OSCE. Based on item response theory examinee performance on a test can be explained by defining examinee characteristics referred to as traits or abilities [20]. The two parameter model estimates the examinees performance or ability on a latent trait with varying item difficulty and discrimination using dichotomous responses. This model simultaneously incorporates difficulty and discrimination of the items or test as a whole and therefore encompasses much more information in items or OSCE stations than does either a Rasch model (single parameter - difficulty) or conventional classical test theory analyses.

The major purpose of the present study, therefore, was to assess the stability of the OSCE stations that were re-used using the 2-facet IRT model. The stability of the OSCE stations should include not only difficulty as in the McKinely and Boulet [16] study but should incorporate discrimination as well as in a 2-facet IRT design. The model we used was based on the assumptions that the sample over different years is from the same universe of examinees, SPs and raters. Therefore, the OSCE stations were kept constant while different samples of examinees, SPs and raters were drawn from their respective universes.

## Method

### Participants

The first sample included international medical graduates that took the (n = 189) OSCE conducted by the Alberta International Medical Graduate Program at Time 1 while the second sample were from (n = 236) examinations one year later (Time 2). There were 89 men (46%) and 100 women (52%) at Time 1 and 100 men (42%) and 136 women (58%) at Time 2. The mean age was 38.8 (SD = 7.3) years at Time 1 and 39.2 (SD = 7.3) years at Time 2. All had received their medical degree (MD, MBBS, MB) from the Medical School listed in the World Health Organization directory of recognized medical institutions and were undergoing assessment in Canada to gain residency positions. The mean years since graduation were 13.0 (range: 0.8 – 31) at Time 1 and 13.4 (range, 0.0 – 32) at Time 2. The majority of the participants were from Asia, Russia and Eastern Europe.

#### Procedures

Several high-stakes objective structured clinical exams (OSCEs) have been employed over the years to assess International Medical Graduates (IMG’s). The Alberta International Medical Graduate Program (AIMGP) was created by the Government of Alberta (Alberta Health and Wellness) in 2001 with a mission to increase the number of International Medical Graduates (IMGs) eligible to practice medicine in the province. The AIMG Program uses a 10-station OSCE and a Multiple Mini Interview format to match qualified IMGs to defined residency positions in Alberta. Each year the AIMG program re-uses 4–6 OSCE stations from previous years with good psychometric indices (e.g., index of dependability > 0.70; item analysis – item difficulty and discrimination).

The OSCE is highly dependent on the standardized patients (SPs) and hence their training is comprehensive. Depending on the number of tracks used an equal number of standardized patients (SPs) are trained for each station. It is ensured during training that SPs trained for specific stations perform similarly so that the candidates get similar experience and error due to SP is minimized. Typically during the exam the SPs stay on their respective stations and in both years none of the SPs had to leave and no alternates were used. SPs do not score the candidates and hence for G studies we used the single facet nested design with SPs and examiners nested within the candidates.

All physicians underwent testing in a 10 station OSCE, six of which were repeated at Time 2. The stations were typical assessments designed to assess a number of clinical skills such as history taking, physical examination, lab data interpretation, diagnoses, case management, etc. One trained physician assessor scored each candidate on a standard checklist where each skill was dichotomously scored as either correct or incorrect. SPs did not score the performance of the candidates. At Time 1 OSCE, eight parallel tracks at two sites were administered and 84 examiners were recruited, with 2 alternate examiners for each site. At Time 2 first OSCE was conducted at two sites with eight parallel tracks and the second was conducted at one site after two weeks. One hundred and seven (107) examiners participated with 2 alternates at each site on both times. All examiners were physicians that had undergone at least 30 minutes training for assessment on their OSCE station. All stations are criterion referenced for pass / fail employing a minimum performance level (MPL) derived by the Ebel method.

### Stations at time 1 and 2

The six stations (Table 1) at both times were A) Post-Cardiac Event Counseling, B) Life Change (Menopause), C) Stomach Pain, D) Fatigue / Depression, E) Hand Injury, and F) Urinary Infection. The following skills were assessed: History taking, physical examination, information sharing, counselling, data interpretation, case management and providing a differential diagnosis.

**Table 1:** Stability of OSCE Stations for Two Years on Item Response Calibrated Discrimination and Difficulty for a 2-Parameter Logistic Model

**OSCE Station**	**Mean Discrimination and Difficulty Time 1**		**Mean Discrimination and Difficulty Time 2**		**Grand Mean Discrimination and Difficulty (T1 and T2)**				**Number Items**
	**Disc**^**†**^	**Diff**^**††**^	**Disc**	**Diff**	**Disc**	**SE**	**Diff**	**SE**	
Station A	.61	-.95	.56	−1.18	.59	.013	−1.01	.229	21
Post-Cardiac Event Counseling									
Station B	.62	−2.26	.60	−2.00	.61	.015	−2.11	.159	14
Life Change (Menopause)									
Station C	.58	-.15	.62	-.51	.60	.019	-.33	.235	24
Stomach Pain									
Station D	.70	-.33	.74	-.60	.72	.010	−47	.195	30
Fatigue / Depression									
Station E	.63*	-.23	.57*	-.40	.60	.011	-.32	.267	24
Hand Injury									
Station F	.57	-.56	.57	-.80	.57	.012	-.68	.266	27
Urinary Infection									

^†^Discrimination, ^††^Difficulty, *p < .05; ^¶^SE = Standard Error.

Reliability was assessed through G-studies. The G-analyses were based on a single facet nested design with SP’s and assessors nested within the candidates that were assessed using the formula (a Type 2 Design: single-facet nested design: [candidate x (rater: station)]) [21]:

(1) $$Ep^{2}=\frac{\mathrm{Candidate}\left(\mathrm{var}\mathrm{iancecomponenet}\right)}{\mathrm{Candidate}\left(\mathrm{var}\mathrm{iancecomponent}\right)+\mathrm{Error}\left(\mathrm{var}\mathrm{iancercomponent}\right)}$$

While this type of design does not allow for the estimation of the interaction effect of assessors and/or standardized patients with candidates, it does allow for the determination of the generalizability coefficient of assessors. As the SPs did not score the candidates, and only the examiners did, nesting SP within examiner is appropriate and these factors are confounded.

#### Analyses

In order to determine the stability of OSCE stations over time employing different sample of IMGs, we employed a 2-parameter model (discrimination, difficulty) IRT analysis. The third possible parameter (guessing) which is frequently used for multiple choice items, was not employed since this was not possible for clinical skills items that are dichotomously scored as correct or not without the possibility of guessing. The same six OSCE stations were used at Time 1 and Time 2 (see Table 1).

Early methods of item parameter estimating techniques such as “pure” maximum likelihood required long tests and large samples (i.e., several thousand examinees) in order to obtain accurate IRT parameter estimates. Recent advances (Mislevy & Bock, 1985) [22] of estimation such as marginal maximum-likelihood (MML) parameter estimation as implemented in XCALIBRE 1.1, can however give reasonable estimates of IRT item parameters which are derived from short tests and small samples of examinees (e.g., one hundred). The IRT analyses were conducted with XCALIBRE (version 1.1) which has the capability for a three-parameter logistic (frequently employed with multiple choice items) IRT model [20]:

(2) $$P\left(u_{g}=1|\theta_{i}\right)=\frac{1}{1+{e^{-}}^{Da_{g}\left(\theta_{i}-b_{g}\right)}}$$

Where: D is the constant 1.702 which approximates the normal ogive

*a*_*g*_ is the item discrimination parameter

*b*_*g* is_ the item difficulty parameter

*c*_*g*_ is the lower asymptote (guessing) parameter

For the two-parameter model, *c*_*g*_ (the lower asymptote for the guessing parameter) is set at zero (guessing is not a parameter in clinical skills assessment).

One-way multivariate analyses of variance (MANOVA) with year as the independent variable and discrimination and difficulty as the dependent variables were conducted for each OSCE station.

Test Characteristic Curves (TCCs) were computed with homogenous items for each year. The pattern of TCC for homogenous and heterogeneous items for all the OSCE stations was similar. We present results from stations A and B for comparison. TCCs were computed for each of the six stations with all items for Time 1 and Time 2 administration. The TCCs simultaneously graphically display the difficulty of the test (station) and the overall discrimination.

## Results

Generalizability coefficients (Ep^2^) employing a single facet nested design G-study ranged from 0.51 to 0.78 (for the varying 6 stations used at both Time 1 and 2). The difficulty and discrimination parameters are summarized in Table 1. The difficulty indices for Station A and B indicate that they are relatively easy stations (difficulty = −1.01, -2.11 respectively) compared to Stations C, D, E, and F (−.33, -.47, -.32, -.68 respectively). The discrimination coefficients for all 6 stations are quite homogeneous (range: .57 to .72). Nearly all of the generalizability coefficients (Ep^2^) of the OSCE stations were in the adequate to good range (> .70).

The results of the one-way MANOVAs are also summarized in Table 1. Only one of these analyses resulted in a significant difference (Station E: Wilks lambda = .869, *F* = 3.39, *p* < .05). Follow-up one way ANOVAs indicated that the significant difference was for discrimination (*p* < .05) but not for difficulty (Table 1). The remaining non-significant results show that the OSCE station results are stable over the 2 years; there are non-significant differences on difficulty for the OSCE stations over the two years and, with the exception of Station E, no differences in discrimination.

**Figure 1:**
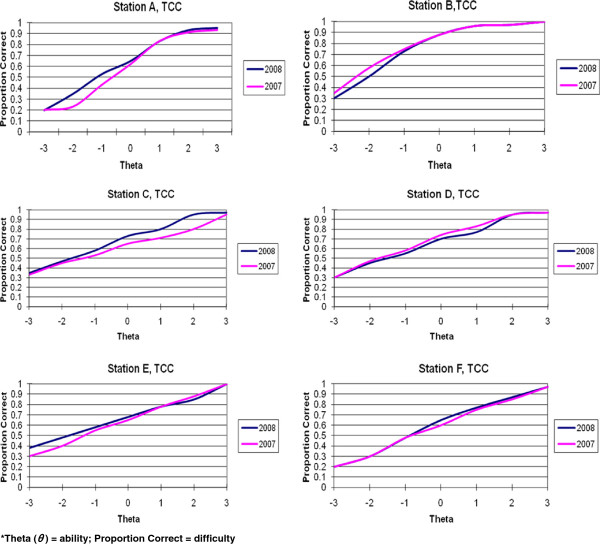
Test Characteristic Curves for Six OSCE Stations in Time 1 and Time 2*. *Theta (*θ* ) = ability; Proportion Correct = difficulty.

The TCCs are depicted in Figure 1. This figure contains a visual representation of the TCCs for each OSCE station (overlapped from Time 1 and Time 2) so that they can be visually compared over the two year period. As can be seen from a close inspection the TCCs, they are very similar from year to year (Time 1 to Time 2) in shape and mean ability levels. Station A produces the closest to the ideal TCC with appropriate difficulty (mean *θ* = −1.01) while Station E results in a linear type TCC.

## Discussion

The main findings of the present study were that: 1) The OSCE stations had acceptable Ep^2^ coefficients for the assessors [21], 2) The OSCE stations assessing clinical skills were stable across different samples when we employed a 2-parameter logistic model analyses, and 3) TCCs yield a similar patern (difficulty and discrimination) when used with OSCE stations assessing the same problem irrespective of the homogeneity of items.

A great deal of effort and research has gone into the study of the reliability of OSCEs over the past 40 or so years [7]. The primary focus, however, has been on the internal consistency of OSCE stations or generalizability (e.g., Ep^2^) of various facets (e.g., raters, SPs, stations, interactions, etc.). Very little work has been done on the systematic study of OSCE stations temporal stability even though stations are very frequently used repeatedly over time even with high stakes exams. In the present paper we developed a method for a comprehensive analysis of temporal stability of OSCE stations employing a 2-parameter logistic model employing an IRT approach. This approach is preferable to classical test theory or generalizability theory for testing the stability of OSCEs as it can simultaneously take into account 2 critically important parameters in the IRT model to derive overall TCCs for comparison [20].

In the present study, we found that items assessing clinical skills remain stable over different samples of examinees that come from the same theoretical population when they are calibrated for difficulty and discrimination. The minor difference in discrimination found for one of the stations likely reflects sampling error. Otherwise, we can remain confident that for the 2-parameter logistic model, OSCE skills assessments are stable for different samples of examinees and examiners.

The TCCs developed with heterogeneous items were similar to curves computed with all items on the checklist. This suggests that clinical skills are contextual and not dependent on checklist items. This result is in concordance with the Baig et al. study which provided empirical evidence for the contextual nature of communication skills [23]. The present results show that irrespective of the heterogeneity of the items/traits, the TCCs showed similar pattern of difficulty and discrimination.

There are some limitations of the present study. While the 6 stations that we employed to study stability were typical assessments designed to assess a number of clinical skills such as history taking, physical examination, lab data interpretation, diagnoses, and case management, we did not attempt to differentiate between different skills such as physical exams, counseling, etc. It may be that one type of clinical skill is more stable than others. The training for the examiners was only 30 minutes which is very brief and could be improved in future research. We also employed IMGs and only two years of data to assess stability. The study should be extended to other sorts of candidates such as medical students, residents, candidates for licensure, etc. Moreover, the stability can be studied over a longer period than 2 administrations of the same OSCE.

## Conclusions

In the present study we employed an IRT model that allows the systematic study of the temporal stability of OSCE stations. Using difficulty and discrimination as dependent variables in a MANOVA design allowed us to precisely determine any differences in 2 stations that may exist. Moreover, we were able to calibrate the 2-parameters on a logistic model and compare the TCC curves for the same stations. Future research should focus on extending our findings to other samples of candidates, raters and skills assessed over longer periods of time. Meanwhile, indicate that OSCE stations have temporal stability even across different candidates and assessors.

## Competing interests

Both authors declare that they have no competing interests’.

## Authors’ contributions

LB collected the data, did the analysis and wrote the first draft, CV supported in the study and analysis and wrote the second draft. Both authors read and approved the final manuscript.

## Pre-publication history

The pre-publication history for this paper can be accessed here:

http://www.biomedcentral.com/1472-6920/12/121/prepub
